# Limited View Tomographic Reconstruction Using a Cascaded Residual Dense Spatial-Channel Attention Network With Projection Data Fidelity Layer

**DOI:** 10.1109/TMI.2021.3066318

**Published:** 2021-06-30

**Authors:** Bo Zhou, S. Kevin Zhou, James S. Duncan, Chi Liu

**Affiliations:** Department of Biomedical Engineering, Yale University, New Haven, CT 06511 USA; Institute of Computing Technology, Chinese Academy of Sciences, Beijing 100190, China; Department of Biomedical Engineering, Yale University, New Haven, CT 06511 USA, and also with the Department of Radiology and Biomedical Imaging, Yale University, New Haven, CT 06511 USA; Department of Biomedical Engineering, Yale University, New Haven, CT 06511 USA, and also with the Department of Radiology and Biomedical Imaging, Yale University, New Haven, CT 06511 USA

**Keywords:** Tomographic reconstruction, cascaded network, projection data fidelity layer, RedSCAN, limited angle, sparse view

## Abstract

Limited view tomographic reconstruction aims to reconstruct a tomographic image from a limited number of projection views arising from sparse view or limited angle acquisitions that reduce radiation dose or shorten scanning time. However, such a reconstruction suffers from severe artifacts due to the incompleteness of sinogram. To derive quality reconstruction, previous methods use UNet-like neural architecturesto directly predict the full view reconstruction from limited view data; but these methods leave the deep network architecture issue largely intact and cannot guarantee the consistency between the sinogram of the reconstructedimage and the acquiredsinogram, leading to a non-ideal reconstruction. In this work, we propose a cascaded residual dense spatial-channel attention network consisting of residual dense spatial-channel attention networks and projection data fidelity layers. We evaluate our methods on two datasets. Our experimental results on AAPM Low Dose CT Grand Challenge datasets demonstrate that our algorithm achieves a consistent and substantial improvement over the existing neural network methods on both limited angle reconstruction and sparse view reconstruction. In addition, our experimental results on Deep Lesion datasets demonstrate that our method is able to generate high-quality reconstruction for 8 major lesion types.

## I. Introduction

Tomography imaging is a non-invasive projection-based imaging technique that visualizes an object’s internal structures and hence finds wide applications in healthcare, security, and industrial settings [1]–[3]. In healthcare, tomography imaging techniques such as medical Computed Tomography (CT) based on x-ray projections, Positron Emission Tomography (PET), and Single-photon Emission Computed Tomography (SPECT) based on gamma-ray projections are indispensable imaging modalities for disease diagnosis and treatment planning. In the traditional CT setting, one assumes access to the measurements that are collected from a full range of view angles of an object. To reduce radiation dose and speed up acquisition, recently it is of increasing interest to develop methods that can recover images when a portion of the projection views is missing, namely limited view tomographic reconstruction. There are two notable sub-problems: limited angle (LA) reconstruction, i.e., when *α* ∈ [0*, α*_*max*_] with *α*_*max*_ *<* 180° for equivalent parallel beam geometry, and sparse view (SV) reconstruction with a view interval larger than normal. Both LA and SV acquisitions can efficiently reduce radiation dose. Using LA acquisition, the scan time can also be drastically reduced by restricting the physical movement of the scan arc. Note that fast acquisition or high temporal resolution is paramount; even a slightly longer scan time can lead to appreciable motion blur and artifact in the image [4], [5].

There are two major factors, namely reconstruction quality and speed that need to be properly considered in designing a tomographic reconstruction algorithm. Currently, Filtered Back Projection (FBP) is widely used as the standard algorithm as it can reconstruct a high-quality image with a fast speed, following an analytical solution. However, FBP assumes the access to the measurements that are collected from a full range of views of an object. Reconstruction using FBP in both LA and SV conditions are highly ill-posed, yielding non-ideal image quality with severe artifacts and high noise. Previous algorithms for tomographic reconstruction under limited view conditions can be classified into two general categories: model-based iterative reconstruction (MBIR) and deep learning based reconstruction (DLR). MBIR can generate images with high quality by minimizing the predefined image domain regularizers and the sampled sinogram inconsistency in an iterative fashion. Common choices of the regularizer include total variation [6], dictionary learning [7], and nonlocal patches [8]. However, MBIR methods are computationally heavy and time-consuming since they rely on repetitive forward- and back-projections. Moreover, using regularization solely based on prior assumptions requires careful hyper-parameter tuning and tends to bias the reconstruction results, especially when under-sampling rate is high.

Recently, deep learning techniques, such as convolutional neural networks (CNNs), have been widely adapted in tomography and demonstrated promising reconstruction performance [9]. Combining MBIR with deep learning, Gupta *et al.* [10] and We *et al.* [11] first proposed to model regularizer in MBIR frameworks with CNNs and Autoencoders. Adler et el. [12] unfolded the optimization procedure of MBIR to an N-stage network to balance the tradeoff between reconstruction and speed. Although improved over traditional MBIR methods, they still suffer from high computational cost with iterative procedures. As an alternative, DLR is often formulated as image post-processing. Jin *et al.* [13] and Chen *et al.* [14] proposed to use UNet [15] and Residual UNet to post-process the noise/artifacts in the sparse-view CT. In [16] and [17], adversarial loss and perceptual loss were used to reinforce the network’s learning. Later, Zhang *et al.* [18] and Han *et al.* [19] proposed to incorporate dense block and wavelet decomposition into UNet for more robust feature learning for reconstruction. Direct sinogram inversion and sinogram completion strategies were also proposed. Lee *et al.* [20] found that synthesizing complete sinogram from sparse view sinogram and then using FBP can also reconstruct high-quality image. Although these methods can be easily applied to raw sinograms or corresponding FBP reconstructed images with relatively low computational cost and low design complexities, they either only applied on image domain that remove artifacts in already reconstructed image or synthesizing complete sinogram from sparse one, and cannot guarantee the sampled sinogram data are preserved. Note that the sampled sinogram data are the original sources that should be kept as identical as possible before and after reconstruction to ensure the high fidelity of reconstructed content. There are also recent ideas of replacing the already-sampled sinogram to the predicted sinogram during the test stage. Anurudh *et al.* [1] proposed to first use a sonogram-to-image auto encoder to predict an initial reconstruction. Then, during the test stage, the reconstruction’s sinogram is partly replaced by the already-sampled sinogram to generate a final reconstruction. However, their method does not guarantee the continuity between the already-sampled sinogram and the predicted sinogram, which may further degrade the final reconstruction, and their method is limited to parallel-beam geometry. Similarly, Huang *et al.* [21] proposed to first use UNet [15] to predict an initial reconstruction. Then, during the test stage, the initial reconstruction is utilized in a TV reconstruction to help the projection data fidelity constraint of unmeasured projection data. However, the final reconstruction quality relies on a high-quality initial reconstruction from UNet’s prediction. In addition, the projection data fidelity constraint of unmeasured projection data is not incorporated in the network design and used only in the separated test stage. On a different note, the network design issue is highly under-explored as a research topic and still limited to UNet-based or auto-encoder architectures [13], [14], [16], [17], [19], [20], [22]. In addition, none of previous works have evaluated the performance under both LA and SV scenarios, and reconstruction evaluation on CT scan with pathological finding are barely performed. While a k-space data consistency layer for MRI fast reconstruction is proposed in [23], [24], projection data consistency layer has not been systematically studied in tomographic reconstruction.

To tackle these limitations, we propose a **Cas**caded **Re**sidual **D**ense **S**patial-**C**hannel **A**ttention **N**etwork (CasRedSCAN) for tomographic reconstruction under limited view conditions. Our CasRedSCAN consisting of Residual Dense Spatial-Channel Attention Network (RedSCAN) and Projection Data Fidelity Layer (PDFL) closely resembles the iterative process in MBIR methods, which allows end-to-end optimization of the reconstruction. Specifically, RedSCAN is the backbone network that is used in each cascade block for de-aliasing the input image. PDFL is concatenated to the RedSCAN output to ensure the prediction’s projection data fidelity while allowing gradient back-propagation. Experiments on limited angle and spare view scans using AAPM Low Dose CT Grand Challenge [25] and DeepLesion dataset [26] demonstrate that our CasRedSCAN can provide high-quality limited view tomographic reconstructions.

## II. Problem Formulation

Let $I\in {\mathbb{C}}^{N}$ represent a 2D tomography image with a size of *N* = *N*_*x*_ *N*_*y*_, and $Q\in {\mathbb{C}}^{M}$ represent its full-view sinogram with *M* projection views. Our problem is to reconstruct *I* from $Q_{u}\in {\mathbb{C}}^{M_{u}}\;\left(\begin{array}{ll} M_{u} & \ll M \end{array}\right)$, where *Q*_*u*_ is the undersampled sinogram of limited views. Here, sinogram data is only measured for lines corresponding to a subset Ω⊂A≜{1,⋯,M}, where A is the full projection set. Denoting G and $G_{u}$ as the full-view and limited-view discretized forward projection operators, the full-view sinogram *Q* and limited-view sinogram *Q*_*u*_ are obtained via Q=GI and $Q_{u}=G_{u}I$, respectively. While FBP provides stable numerical implementation of pseudo-inverse for *Q*, applying FBP to *Q*_*u*_ in the limited view conditions yields reconstructed *I*_*u*_ with severe artifacts.

Previous works of MBIR propose to solve *I* by

(1) $$\underset{I}{\min}\left[T(I)+\lambda {\left\|G_{u}I-Q_{u}\right\|}_{n}^{n}\right],$$

where T is the regularizer and $\|\cdot {\|}_{n}^{n}$ is the projection data fidelity constraint [6], [27]. Previous deep learning-based, post-processing methods utilize deep networks, denoted as P with parameters *θ*, to estimate the full-view reconstructed image $P\left(I_{u};\theta \right)$ by training P on (*I*_*u*_*, I*_*gt*_) pairs, where *I*_*gt*_ is the full-view reconstruction ground truth. However, these methods only consider a subsequent regularization of the initial solution *I*_*u*_ similar to the functionality of T(⋅) in MBIR, and omit the projection data fidelity constraint of ${\left\|G_{u}I-Q_{u}\right\|}_{n}^{n}$. One should force reconstruction *I* to be well-approximated by the CNN reconstruction and ensure the consistency of acquired data in the projection domain by:

(2) $$\underset{I}{\min}\left[{\left\|I-P\left(I_{u};\theta \right)\right\|}_{2}^{2}+\lambda {\left\|G_{u}I-Q_{u}\right\|}_{2}^{2}\right],$$

However, it is not feasible to directly optimize the above equation since the deep network reconstruction and the projection data fidelity terms are independent. Specifically, as deep network P only operates in the image domain, P is trained to reconstruct the full-view image without prior knowledge of the already acquired data in the projection domain. Similar to the MRI k-space data fidelity [23], given a portion of already acquired projection data from limited-view acquisitions, the deep network should be discouraged from changing the already acquired projection data up to the level of acquisition noise. Incorporating the projection data fidelity in the network design could potentially better preserve the image content and lead to a better reconstruction. In this work, we propose a projection data fidelity layer (PDFL) embedded in a cascade network for full-view reconstruction. With PDFL in our cascade network, the reconstruction output from our network is now conditioned on both network parameter *θ* and limited-view projection data Ω:

(3) $$I_{rec}=P\left(I_{u};\theta ,\Omega \right)$$

Then, given the training data pairs of (*I*_*u*_*, I*_*gt*_), we can train our network by minimizing the L2 loss function:

(4) $$L={\left\|P\left(I_{u};\theta ,\Omega \right)-I_{gt}\right\|}_{2}^{2}$$

Details of our PDFL and cascade network are explained in Section III and Section IV, respectively.

## III. Projection Data Fidelity Layer

Let G and $G_{fbp}$ be forward projection (FP) layer and filtered back-projection (FBP) layer, respectively. The projection data of the image reconstruction by a deep network can be formulated as: $S_{cnn}=GI_{cnn}=GP\left(I_{u};\theta \right)$, where *S*_*cnn*_(*i*) is the *i*-th projection data entry. Similarly, we denote the already acquired projection data as *S*_*u*_, where *S*_*u*_ has identical size to *S*_*cnn*_ and the *i*-th projection data entry *S*_*u*_(*i*) is all zeros when *i* ∉ Ω. Then, we can write a closed-form solution for the second term in Eq.(2) as:

(5) $$S_{rec}(i)=\{\begin{array}{cc} \frac{\lambda S_{cnn}(i)+S_{u}(i)}{\lambda +1} & \;\text{if}\;i\in \Omega \\ S_{cnn}(i) & \;\text{if}\;i\notin \Omega \end{array}$$

where *S*_*rec*_ is the reconstructed sinogram, which is updated by the projection data fidelity. Then, the image can be reconstructed via filtered back projection, that is, $I_{rec}=G_{fbp}S_{rec}$. To elaborate, when the *i*-th projection data is not acquired, we directly estimates the *i*-th projection data from the projection data of the deep network’s output. Otherwise, the *i*-th projection data is a linear combination of the acquired projection data and projection data of the deep network’s output, regularized by noise level parameter *λ*. Assuming noiseless sinogram acquisition, i.e. *λ* = 0, we simply replaces the *i*-th predicted projection data by the acquired projection data.

### A. Forward Projection Layer

Our FP layer G is a differentiable layer implemented with fan-beam geometry, allowing gradient back-propagation while projecting the image into sinogram. In this work, we consider fan-beam geometry with arc detector [28]. Assuming the distance between x-ray source and the gantry rotation center as *D*, the forward pass of the FP layer can be written as:

(6) $$S_{fan}(\gamma ,\beta )=\iint_{{\mathbb{R}}^{2}}I(x,y)\delta [D\;\sin(\gamma )-x\;\sin(\beta -\gamma )\;\;-y\cos(\beta -\gamma )]dxdy$$

where a fan-beam sinogram *S*_*f an*_(*γ, β*) is generated. *β* means the detector rotation angle, and *γ* means the angle between central projection line and detector projection line. In the backward path of G, the loss in the sinogram domain should be aggregated and back-projected to the image domain. Thus, we define the derivative of G with respect to the input image *I* as the filtered back-projection operation $G_{fbp}$ (discussed in Section III-B).

### B. Filtered Back-Projection Layer

Our FBP layer $G_{fbp}$ is also a differentiable layer implemented with fan-beam geometry, allowing gradient back-propagation while reconstructing the image from sinogram. Similar to above, assuming the distance between x-ray source and the gantry rotation center as *D*, we have a fan-beam sinogram *S*_*f an*_(*γ, β*), where *β* is the detector rotation angle and *γ* is the angle between central projection line and detector projection line. Our FBP layer consists of three modules: i) parallel-beam conversion module, ii) filtering module, and iii) back-projection module.

**Parallel-beam conversion module** converts the fan-beam sinogram *S*_*f an*_(*γ, β*) to parallel-beam sinogram *S*_*para*_(*ρ, α*) via:

(7) $$\{\begin{array}{l} \alpha =\beta +\gamma ,\\ \rho =D\;\sin\gamma . \end{array}$$

where the change of variable is implemented by grid sampling^1^ in (*ρ, α*), which allows gradient back-propagation.

**Filtering module** applies the filtering to the converted sinogram *S*_*para*_ in the Fourier domain:

(8) $$\hat{S}=T_{\rho }^{-1}\left\{|\omega |\cdot T_{\rho }\left\{S_{para\;}(\rho ,\alpha )\right\}\right\}$$

where *T*_*ρ*_ and $T_{\rho }^{-1}$ are the discrete Fourier transform and inverse discrete Fourier transform along the detector dimension *ρ*, respectively.^2^ *ω* is the window function and we used Ram-Lak in this work.

**Back-projection module** back-projects the filtered parallel-beam sinogram $\hat{S}$ to the image domain for every projection angle *α* via:

(9) $$I(x,y)=\int_{0}^{2\pi }\hat{S}(x\;\cos\;\alpha +y\;\sin\;\alpha ,\alpha )d\alpha \;\;\approx \Delta \alpha \sum_{i}\hat{S}\left(x\;\cos\;\alpha_{i}+y\;\sin\;\alpha_{i},\alpha_{i}\right)$$

where we parallelize the back-projection operation,^3^ such that the reconstruction can be efficiently computed. In the backward path of $G_{fbp}$, the loss in the image domain should be aggregated and projected to the sinogram domain. Thus, we define the derivative of $G_{fbp}$ with respect to the input sinogram *S*_*f an*_ as the forward projection operation G (discussed in Section III-A).

Here, we use pixel-driven algorithm for our implementation of forward projection and back-projection [29].

### C. Forward and Backward Pass

Our Projection Data Fidelity Layer (PDFL) consists of three operations: i) forward project G, ii) the projection data fidelity of Eq.(5), and iii) the FBP layer $G_{fbp}$. The projection data fidelity of Eq.(5) can be formulated in matrix form as:

(10) $$DS_{cnn}+\frac{1}{\lambda +1}S_{u}$$

where $D=diag\left(e_{1},e_{2},\cdots ,e_{M}\right)$ with:

(11) $$e_{M}=\{\begin{array}{ll} \frac{\lambda }{1+\lambda }, & \text{ when }i\in \Omega ,\\ 1, & \text{ when }i\notin \Omega \end{array}$$

Then, our PDFL combines the three operations discussed above. Specifically, the forward pass of PDFL can be writtern as:

(12) $$P_{PDFL}\left(I_{cnn},S_{u}\right)=G_{fbp}\left(DGI_{cnn}+\frac{1}{\lambda +1}S_{u}\right)\;\;=G_{fbp}DGI_{cnn}+\frac{1}{\lambda +1}G_{fbp}S_{u}$$

where *I*_*cnn*_ is the image predicted from an image-domain deep network and is the input of our PDFL. The output of PDFL is an image with projection data fidelity from limited-view projection data *S*_*u*_. Assuming low noise level, we set *λ* = 0.001 (analyzed in Section V-C.4). Given the forward pass of Eq.(12), the gradient of the PDFL with respect to the input *I*_*cnn*_ can thus be written as:

(13) $$\frac{\partial P_{PDFL}}{\partial I_{cnn}}=G_{fbp}DG$$

which is defined for our PDFL’s backward pass. There is no learnable parameter in our PDFL.

## IV. Cascaded Residual Dense Spatial-Channel Attention Network

Previous MBIR methods solve the optimization problem in Eq.(1) for CT reconstruction by switching the de-aliasing step and the projection data fidelity step back and forth until convergence. However, in many previous deep-learning based reconstruction methods [13], [18], [19], they use single-step deep networks for de-aliasing and reconstruction. Unfortunately, a trained single-step network cannot be used for iterative de-aliasing, since iteratively applying single-step network de-aliasing does not guarantee to converge to a reasonable reconstruction. Moreover, single-step deep networks with limited de-alising capability are prone to issues, such as over-fitting. Therefore, it is desirable to have a network structure that is able to iteratively de-alias the image using a deep network with sufficient de-aliasing capability, while preserving the projection data fidelity. Here, we propose a cascaded network structure, called CasRedSCAN, with basic units of Residual Dense Spatial-Channel Attention Network (RedSCAN) and PDFL.

**Fig. 1.**
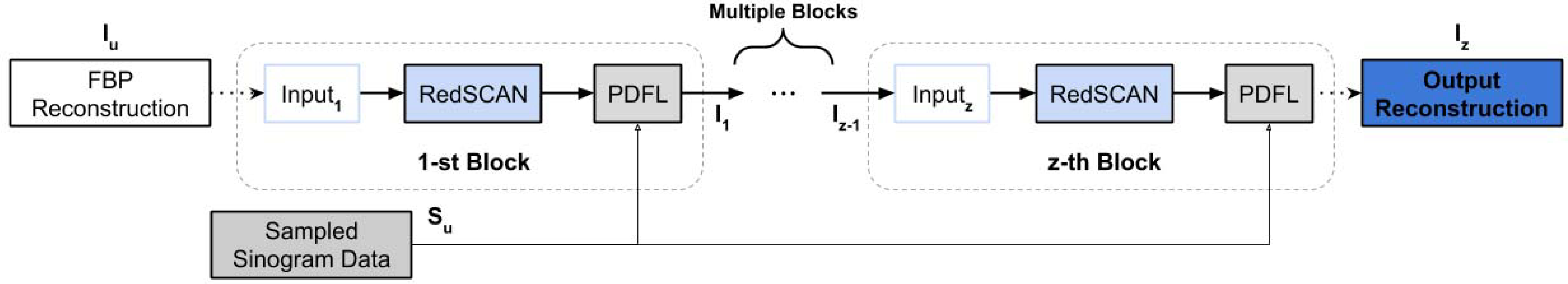
The architecture of our CasRedSCAN. Each block consists of a RedSCAN (blue) and a PDFL (gray).

Similar to the process of MBIR that alternates between the de-aliasing step and the projection data fidelity step, our CasRedSCAN also alternates between the RedSCAN and PDFL, as illustrated in Figure 1. With the initial FBP reconstruction image inputted into the first RedSCAN, the de-aliasing output is fed into the first PDFL. Then, the PDFL output is fed into the second RedSCAN+PDFL block. The same procedure is iterated a fix number of times for a final reconstruction output *I*_*z*_. The loss function can thus be formulated as:

(14) $$L={\left\|P_{CasRedSCAN\;}\left(I_{u};\theta ,S_{u}\right)-I_{gt}\right\|}_{2}^{2},$$

where *I*_*u*_ is initial FBP reconstruction. *θ* is the RedSCAN network parameters. *S*_*u*_ is the limited-view sinogram data. *I*_*gt*_ is the ground truth reconstruction from full-view sinogram data. The algorithm is summarized in Algorithm 1. In our implementation, all the RedSCAN shared the same network parameters in CasRedSCAN, thus maintaining nearly the same model size as compared to the single-step RedSCAN.

### A. Residual Dense Spatial-Channel Attention Network

**Fig. 2.**
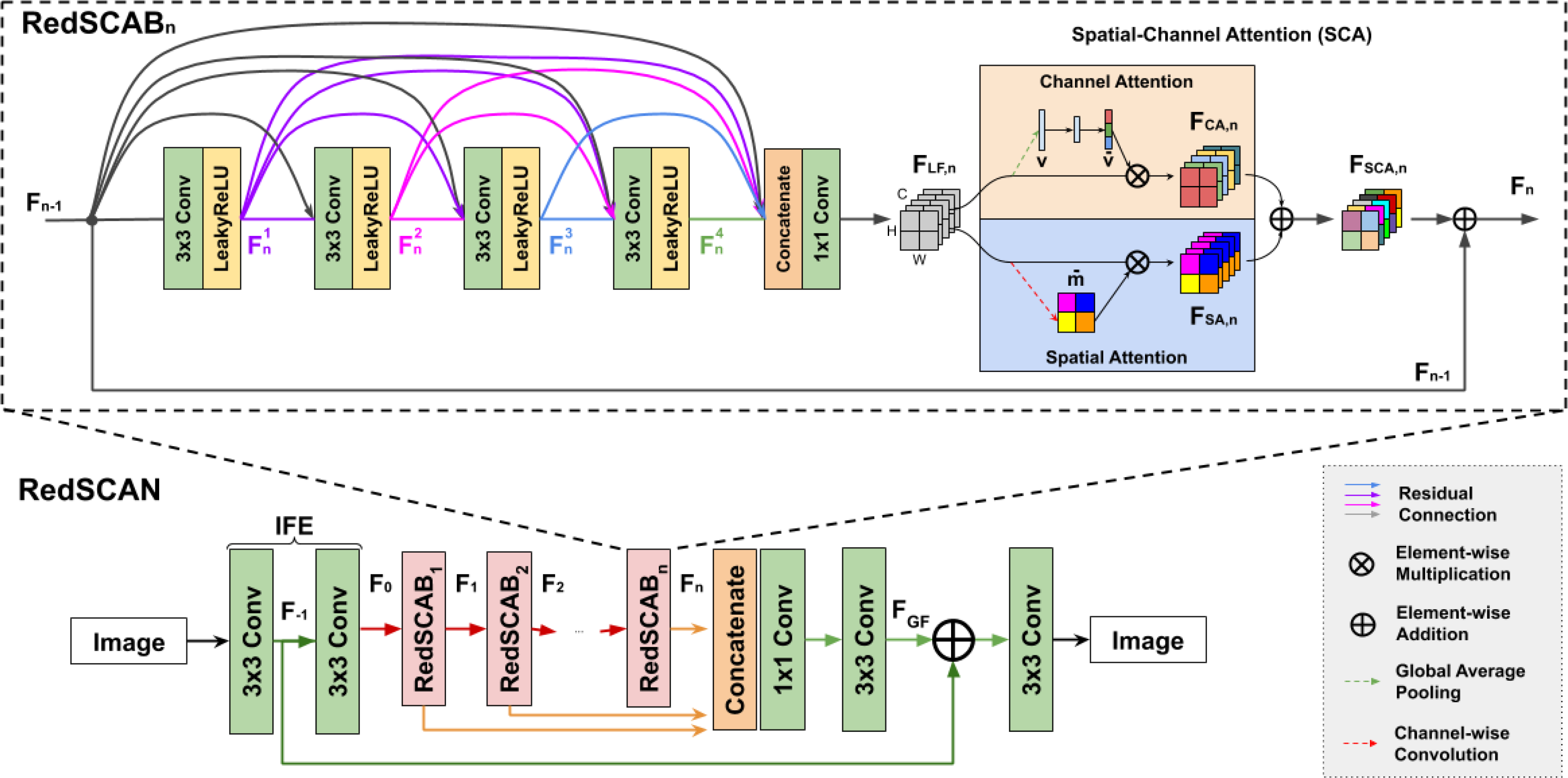
The architecture of our residual dense spatial-channel attention network (RedSCAN), which are used in both the recurrent image reconstruction blocks in Figure 1.

Our RedSCAN consists of three key components, including initial feature extraction (IFE) using two 3×3 convolution layers, multiple Residual Dense Spatial-Channel Attention Block (RedSCAB) followed by global feature fusion, and global residual learning. The network architecture is demonstrated in Figure 2.

Let $P_{IFE_{1}}$ and $P_{IFE_{2}}$ be the first and second convolutional operations in IFE, we first extract $F_{-1}=P_{IFE_{1}}\left(I_{u}\right)$ for global residual learning, and $F_{0}=P_{IFE_{2}}\left(F_{-1}\right)$ for feeding into RedSCAB. Assuming we have *n* RedSCABs, the *n*-th output *F*_*n*_ can thus be written as:

(15) $$F_{n}=P_{RedSCAB_{n}}\left(F_{n-1}\right),$$

where $P_{RedSCAB_{n}}$ represents the n-th RedSCAB operation (*n* ≥ 1). Given the extracted local features from a set of RedSCAB, we apply our global feature fusion (GFF) to extract the global feature:

(16) $$F_{GF}=P_{GFF}\left(\left\{F_{1},F_{2},\ldots ,F_{n}\right\}\right),$$

where {} means concatenation along feature channel and our global feature fusion function $P_{GFF}$ consists of a 1 × 1 and 3 × 3 convolution layers to fuse the extracted local features from different levels of RedSCAB. The GFF output is used as input for our global residual learning:

(17) $$I=P_{final\;}\left(F_{GF}+F_{-1}\right),$$

The element-wise addition of global feature and initial feature are fed into our final 3×3 convolution layer for unregularized output. In our experiment, we set the size of IFE feature channel to 32.

**Algorithm 1:** Cascaded Residual Dense Spatial-Channel Attention Network

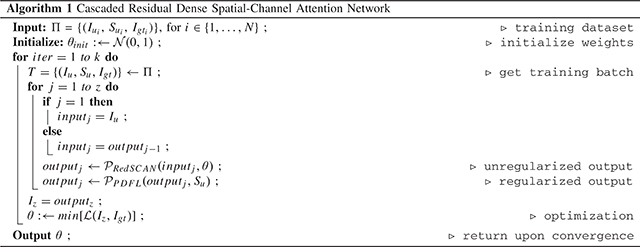

**Residual Dense Spatial-Channel Block** contains four densely connected convolution layers, local feature fusion, local residual connection, and spatial-channel attention. In the *n*-th RedSCAB, the *t*-th convolution output is:

(18) $$F_{n}^{t}=H_{n}^{t}\left\{F_{n-1},F_{n}^{1},\ldots ,F_{n}^{t-1}\right\},$$

where $H_{n}^{t}$ denotes the *t*-th convolution followed by Leaky-ReLU in the *n*-th RedSCAB, {} means concatenation along feature channel, and the number of convolution *t* ≤ 4. Then, we apply our local feature fusion (LFF), a 1 × 1 convolution layer, to fuse the output from the last RedSCAB and all convolution layers in current RedSCAB. Thus, the LFF output can be expressed as:

(19) $$F_{LF,n}=P_{LFF,n}\left(\left\{F_{n-1},F_{n}^{1},F_{n}^{2},F_{n}^{3},F_{n}^{4}\right\}\right),$$

where $P_{LFF,n}$ denotes the LFF operation. Then, it is fed into our Spatial-Channel Attention (SCA) module with two branches to re-weigh channel-wise features and spatial-wise features, as illustrated in Figure 2. The channel attention output *F*_*C A,n*_ and spatial attention output *F*_*SA,n*_ are fused together via *F*_*SC A,n*_ = *F*_*CA,n*_ + *F*_*SA,n*_. Finally, we apply the local residual learning to SCA output by adding the residual connection from RedSCAB input, generating the *n*-th RedSCAB output:

(20) $$F_{n}=F_{SCA,n}+F_{n-1}$$

In our experiment, we set the number of RedSCAB to 5.

**Spatial-Channel Attention** contains two Squeeze-and-Excitation branches for Channel Attention (CA) and Spatial Attention (SA), respectively [30], [31]. Traditional CNNs treat channel-wise features and spatial-wise features equally. However, in an image reconstruction task, it is desirable to have the network focus more on informative features by acknowledging both the channel-wise feature interdependence and the spatial-wise contextual interdependence. The CA and SA structures are illustrated in orange and blue boxes in Figure 2, respectively.

For CA, similar to [30], we spatial-wise squeeze the input feature map using global average pooling, where the feature map is formulated as *F* = [*f*_1_*, f*_2_, …, *f*_*C*_] here with $f_{n}\in {\mathbb{R}}^{H\times W}$ denoting the individual feature channel. We flatten the global average pooling output, generating $v\in {\mathbb{R}}^{C}$ with its *z*-th element:

(21) $$v_{z}=\frac{1}{H\times W}\sum_{i}^{H}\sum_{j}^{W}f_{z}(i,j)$$

where vector *υ* embeds the spatial-wise global information. Then, *υ* is fed into two fully connected layers with weights of $w_{1}\in {\mathbb{R}}^{\frac{C}{2}\times C}$ and $w_{2}\in {\mathbb{R}}^{C\times \frac{C}{2}}$, producing the channel-wise calibration vector:

(22) $$\hat{v}=\sigma \left(w_{2}\eta \left(w_{1}v\right)\right)$$

where *η* and *σ* are the ReLU and Sigmoid activation function, respectively. The calibration vector is applied to the input feature map using channel-wise multiplication:

(23) $${\hat{F}}_{CA}=\left[f_{1}{\hat{v}}_{1},f_{2}{\hat{v}}_{2},\ldots ,f_{C}{\hat{v}}_{C}\right]$$

where ${\hat{v}}_{i}$ indicates the importance of the *i*-th feature channel and lies in [0, 1]. With CA embedded into our network, the calibration vector adaptively learns to emphasize the important feature channels while plays down the others.

In SA, we formulate our feature map as *F* = [*f*^1,1^, …, *f*^*i,j*^, …, *f*^*H,W*^], where $f^{i,j}\in {\mathbb{R}}^{C}$ indicates the feature at spatial location (*i, j*) with *i* ∈ {1, …, *H*} and *j* ∈ {1, …, *W*}. We channel-wise squeeze the input feature map using a convolutional kernel with weights of $w_{3}\in {\mathbb{R}}^{1\times 1\times C\times 1}$, generating a volume tensor $m=w_{3}⊛F$ with $m\in {\mathbb{R}}^{H\times W}$. Each *f*^*i,j*^ is a linear combination of all feature channels at spatial location (*i, j*). Then, the spatial-wise calibration volume that lies in [0, 1] can be written as:

(24) $$\hat{m}=\sigma (m)=\sigma \left(w_{3}⊛F\right)$$

where *σ* is the sigmoid activation function. Applying the calibration volume to the input feature map, we have:

(25) $${\hat{F}}_{SA}=\left[f^{1,1}{\hat{m}}^{1,1},\ldots ,f^{i,j}{\hat{m}}^{i,j},\ldots ,f^{H,W}{\hat{m}}^{H,W}\right]$$

where the calibration parameter ${\hat{m}}^{i,j}$ provides the relative importance of a spatial information of a given feature map. Similarly, with SA embedded into our network, the calibration volume learns to stress the most important spatial locations while ignores the irrelevant ones.

Finally, channel-wise calibration and spatial-wise calibration are combined via element-wise addition operation $F_{SCA}={\hat{F}}_{SA}+{\hat{F}}_{CA}$. With the two branch fusion, features at (*i, j, c*) possess high activation only when they receive high activation from both SA and CA. Our SCA encourages the networks to re-calibrate the feature map such that more accurate and relevant feature maps can be learned.

## V. Experiments and Results

### A. Data Preparation and Training

We used two large-scale dataset for our experiments. In our first dataset, we collected 10 whole body CT scans from the AAPM Low Dose CT Grand Challenge [25]. Each 3D scan contains 318 ~ 856 2D slices covering a range of anatomical regions from chest to abdomen to pelvis. From the AAPM dataset, the 2D dataset of 3397 images without lesion are split patient-wise into 1834 training images, 428 validation images, and 1135 test images. To evaluate the reconstruction performance on CT image with important pathological findings, in our second dataset, we collected 2900 2D CT slices from the DeepLesion dataset [32], which consists of 8 different lesion types (bone:240, liver:380, lung:380, kidney:380, mediastinum:380, abdominal:380, pelvis:380, soft-tissue:380). We split the DeepLesion 2D dataset into 1960 training images (110 for bone, 250 for each of the rest lesion types), 300 validation images (50 slices for each lesion types), 640 test images (80 slices for each lesion types). All images are resized to 256 × 256. We combined two dataset for training and testing.

Similar to the CT projection simulation in [33], we assume an equi-angular fan-beam projection geometry. A 120 *kV p* polyenergetic x-ray source is simulated. To simulated Poisson noise in the sinogram, we assume the incident x-ray contains 2 × 10^7^ photons. The distance between the x-ray source and the rotation center is set to 39.7 *cm*. There are 439 detector bins in a row and each image consists of 256 × 256 pixels. For each image, the fully sampled sinogram data *S* was generated via 360 projection views uniformly spaced between 0 and 360 degrees. In sparse view experiments, we uniformly sampled 180, 90, and 60 projection views from the 360 projection views to form *S*_*u*_, mimicking 2, 4, and 6 fold radiation dose reduction. In limited angle experiments, we sampled 90, 120, and 150 (out of the 360 total) projection views that lies within 0 − 90, 0 − 120, and 0 − 150 degrees for our *S*_*u*_. The reconstructed image *I* and *I*_*u*_ were obtained by applying FBP to *S* and *S*_*u*_, respectively.

We implemented our CasRedSCAN in Pytorch,^4^ and trained it on an NVIDIA Quadro RTX 8000 GPU with 48G memory. The Adam solver [34] was used to optimize our models with a momentum of 0.99 and a 0.0005 learning rate. We used a batch size of 4 during training.

### B. Experimental Results

For quantitative evaluation, both SV and LA results were evaluated using Peak Signal-to-Noise Ratio (PSNR), Structural Similarity Index (SSIM), and Root Mean Square Error (RMSE) by comparing the synthetic SV and LV reconstructions to the ground truth reconstruction from FBP of fully sampled sinogram. For comparative study, we compared our results on both SV and LA tasks against: 1) image-to-image translation-based methods, including the combination of Densenet and Deconvolution (DDNet) [18], Framing UNet (FUNet) [19], FBPNet [13], and 2) deep learning-based methods with projection data fidelity used in the test stage, including DCAR [21] and CTNet [1].

**Fig. 3.**
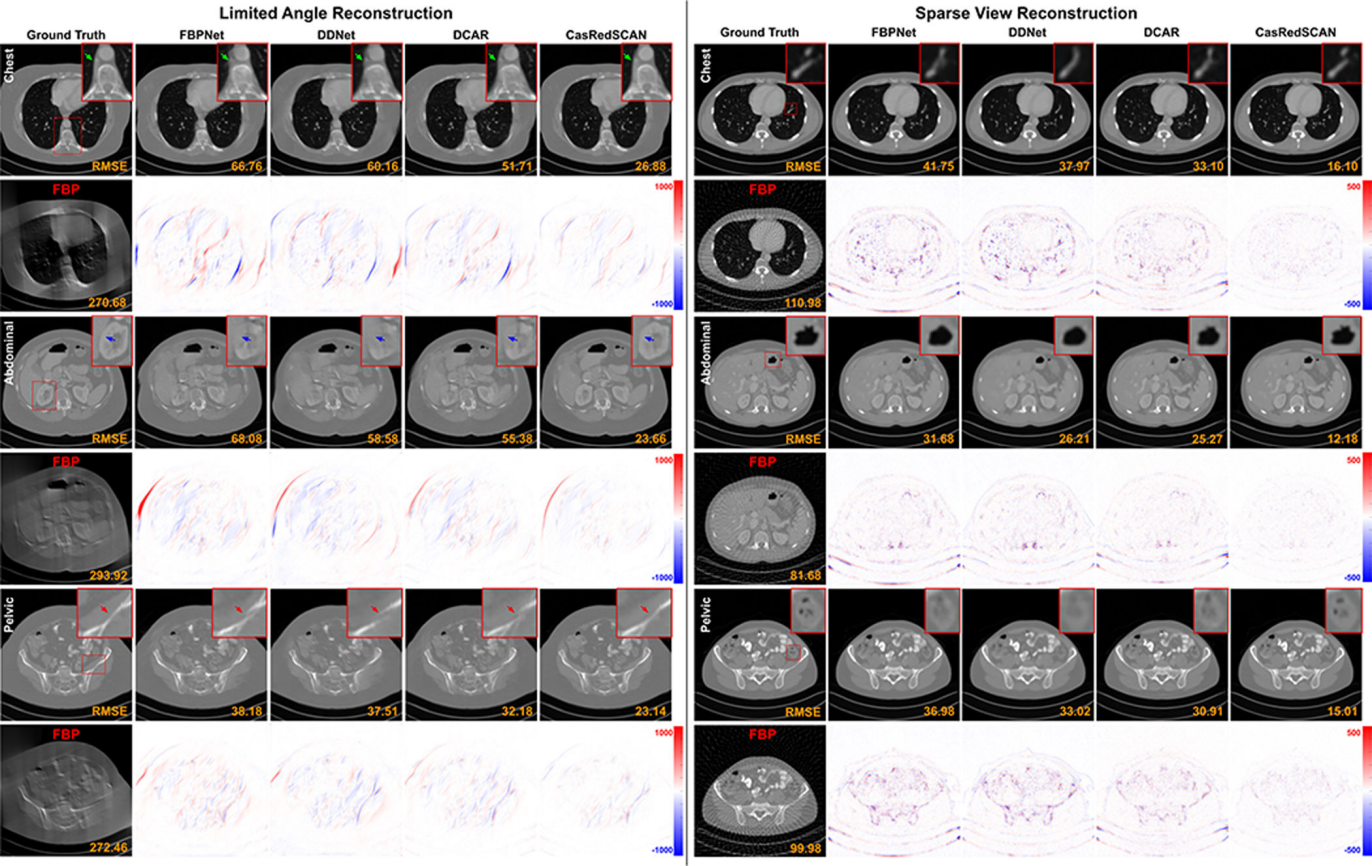
Comparison of **limited angle reconstructions** (120° limited angle) and **sparse view reconstructions** (1/4 downsampling) in chest, abdominal, and pelvic CT scans along with error maps. In our LA chest reconstruction, important arterial structure (green arrows) is better preserved using our CasRedSCAN. Similarly for kidney boundary (blue arrows) in the abdominal reconstruction. The corresponding RMSE is indicated at the bottom. The display window is [−1000 1000] HU.

The qualitative comparison of different limited angle reconstruction methods with AAPM dataset is shown in Figure 3. As we can observe in chest region, previous methods have difficulties in reconstructing small anatomical structure, i.e. arteries. Similarly, with crowded organs in abdominal region, the organ boundaries are challenging to recover by previous methods along with additional patient boundary artifacts. Our CasRedSCAN with advanced network design and projection data fidelity constraint can provide superior limited angle reconstruction in terms of organ boundary recovery, small structure recovery, and boundary artifact elimination. Table VI outlines the quantitative comparison of different methods on limited angle reconstruction with AAPM dataset. Compared to the best previous method’s performance of DCAR [21], we improve SSIM from 0.970 to 0.983 and reduce RMSE from 39.1 to 26.1 for 120° setup, respectively.

**Fig. 4.**
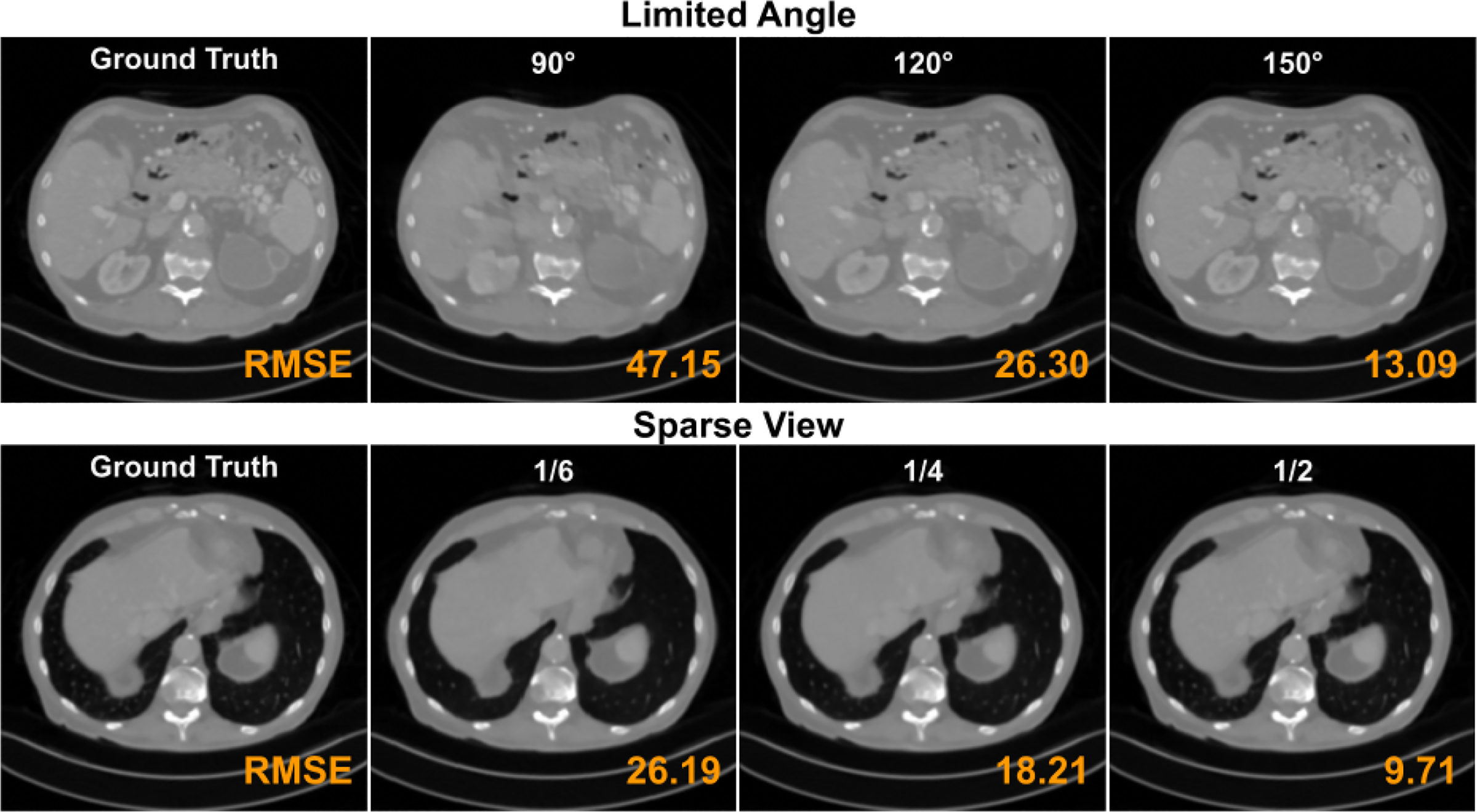
**Limited angle reconstructions** and **sparse view reconstructions** at different limited angle settings and downsampling ratio settings. The display window is [−1000 1000] HU.

The qualitative comparison of different sparse view reconstruction methods with AAPM datset is also shown in Figure 3. Similar to the observations from limited angle experiments above, our CasRedSCAN yields high-quality reconstruction in crowded soft tissue area with fine details. As evidenced in Table VI, our CasRedSCAN achieves the best results among various previous methods. Compared to the best previous method’s performance of DCAR [21], we improve SSIM from 0.973 to 0.989 and reduce RMSE 26.3 to 14.4 for 1*/*4 setup, respectively. Figure 4 shows the limited angle reconstructions and sparse view reconstructions from our CasRedSCAN at different settings.

**Fig. 5.**
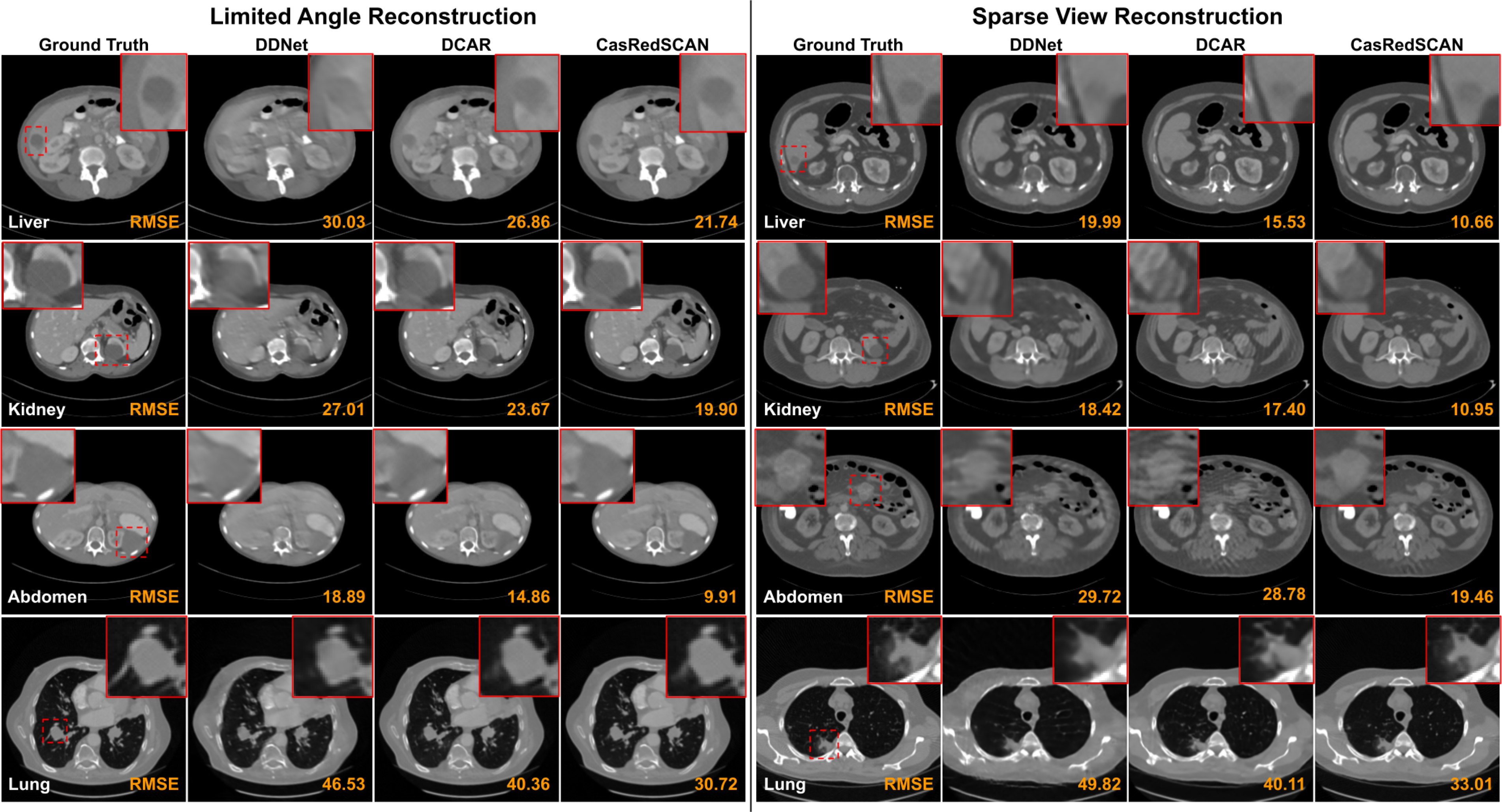
Comparison of **limited angle reconstructions** (120° limited angle) and **sparse view reconstructions** (1/4 downsampling) in CT scans with lesions. The lesion region zoom-in views are shown on the top. The display window of liver, kidney, and abdomen CT is [−300 500] HU. The display window of lung CT is [−1000 1000] HU.

**Fig. 6.**
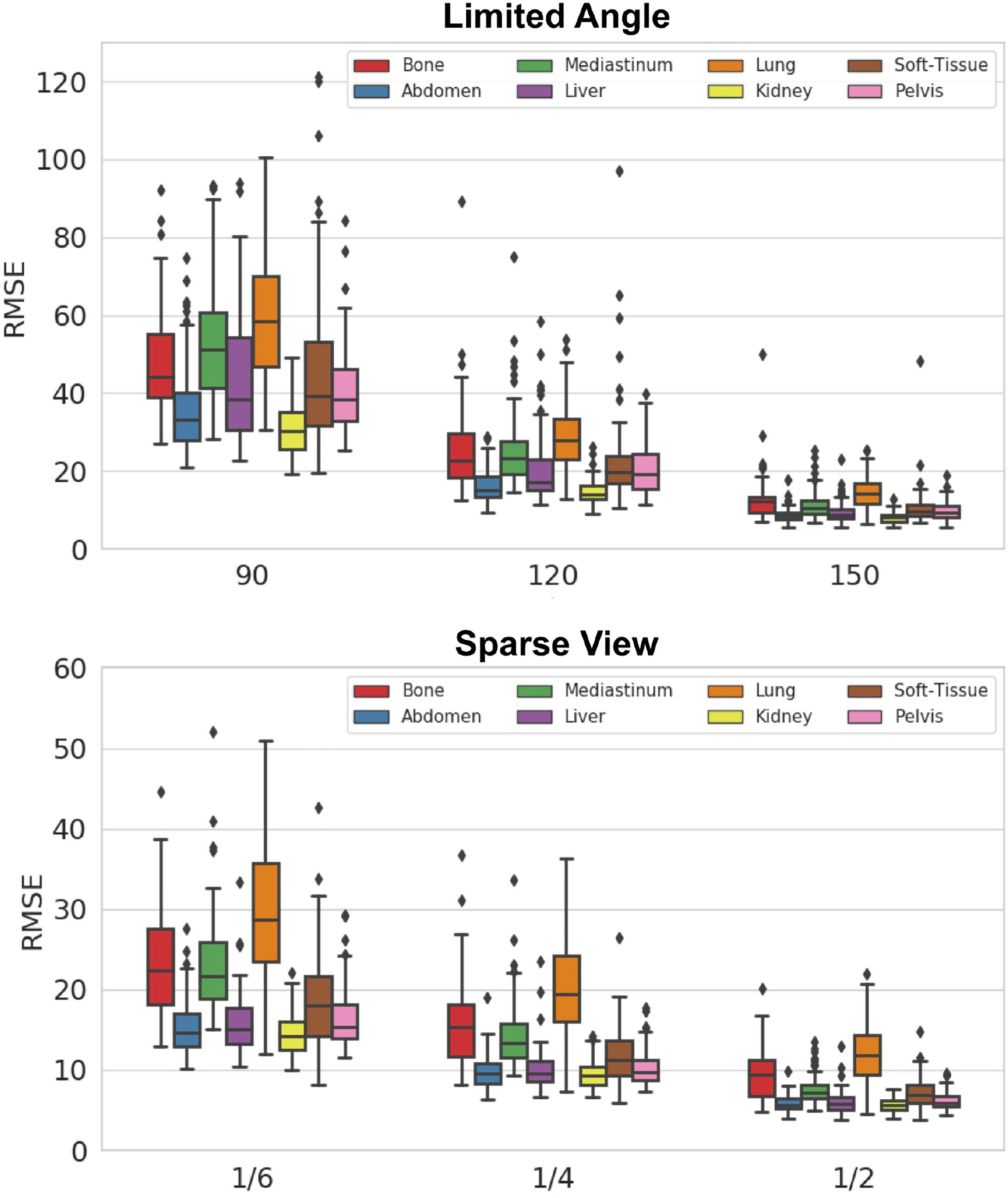
Comparison of **limited angle** and **sparse view** results on CT images with 8 tumor types under different limited view settings.

As CT scan is often used for disease diagnosis, we also evaluated the reconstruction performance on CT images with 8 different lesion types. Figure 5 illustrates the qualitative comparison of various limited angle and sparse view reconstruction methods on 4 major lesion types. As we can observe, the liver lesion and kidney lesion are hard to recover by previous methods because these lesions have low contrast to the soft-tissue background, and their visualization are further degraded by the limited angle artifacts. Similarly, the lung lesion are also challenging to recover by previous methods due to their complex lesion texture. However, our CasRedSCAN can provide superior recovery of the shape and texture of the lesion even under these difficult conditions. For example, our liver and kidney reconstructions on the last column can provide clear lesion boundary which is critical for lesion progression assessment. The lung bronchi that originally diminished on FBP reconstruction can also be better recovered by our CasRedSCAN. Table II summarizes the reconstruction performance on CT images with 8 different lesion types. For 120° limited angle reconstruction, our CasRedSCAN achieves RMSE *<* 30 HU across all 8 lesion types which consistently outperforms previous reconstruction methods. Similarly, for 1*/*4 sparse view reconstruction, our CasRedSCAN achieves the lowest RMSE across all 8 lesion types as compared to previous reconstruction methods. Performance comparison of our CasRedSCAN under different limited angle and sparse view settings on 8 different tumor types are illustrated in Figure 6. Our CasRedSCAN is able to keep the RMSE below 20 for limited angle reconstructions (150°) and sparse angle reconstructions (1*/*2) with different tumor types. However, the RMSE increases as the limited angle reduces or the sparse view undersampling rate increases.

### C. Ablation Studies

#### 1) Number of Cascade:

**Fig. 7.**
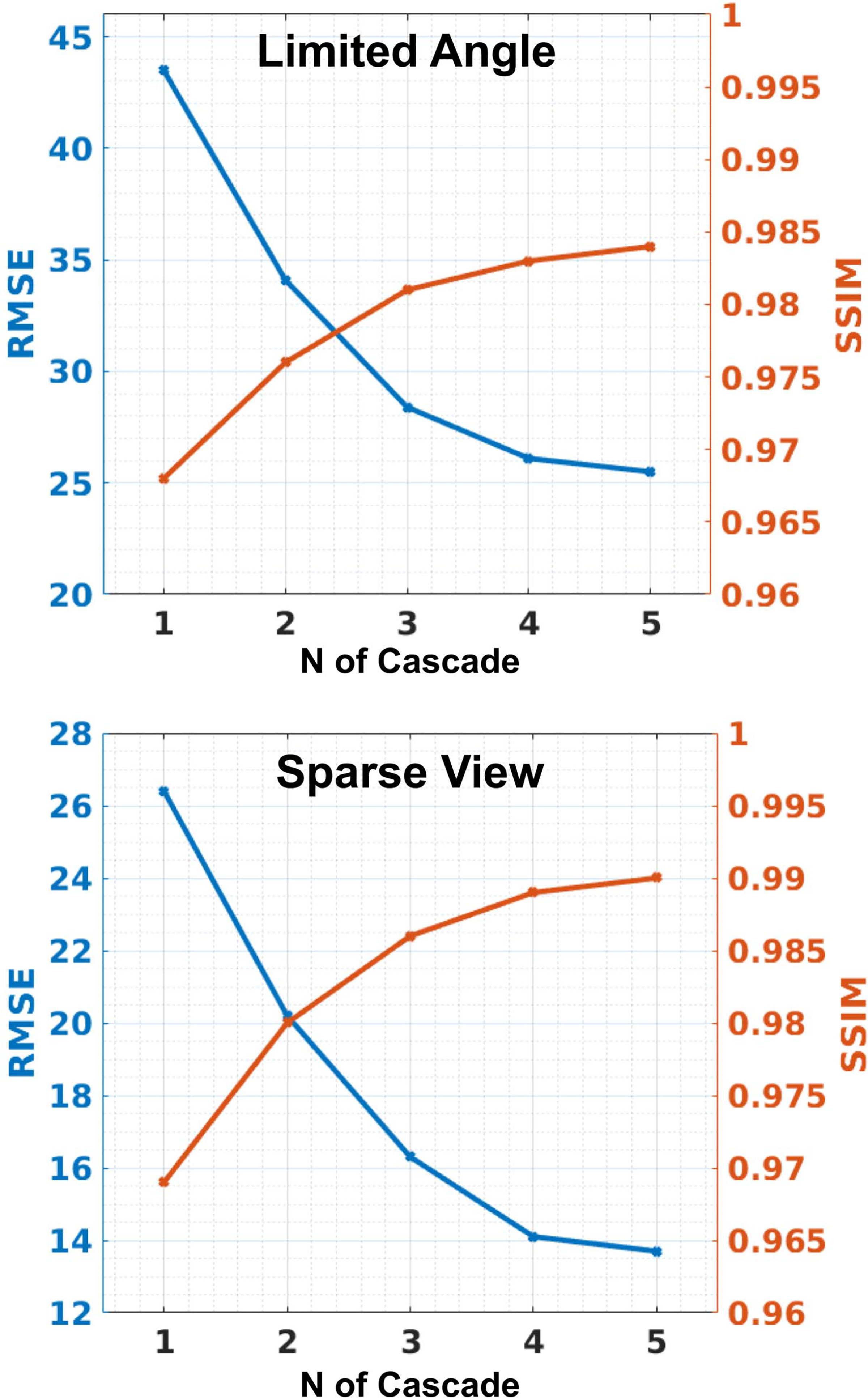
The effect of increasing the number of cascade blocks (Z) in our CasRedSCAN for **limited angle reconstructions** (120° limited angle) and **sparse view reconstructions** (1/4 downsampling).

The number of cascade block can be flexibly adjusted in our CasRedSCAN. We analyzed the effect of increasing the number of cascade blocks in our CasRedSCAN. The result is summarized in Figure 7 and evaluated using AAPM dataset. As we can observe, using more cascade blocks boosts the reconstruction performance, while the rate of improvement starts to converge after the number of blocks reaches 3. In LA, increasing the number of cascade from 4 to 5 only increase SSIM by less than 0.002 and reduce RMSE by less than 2 in average. Similar observation can be found in SV.

#### 2) Attention Mechanism:

Two attention mechanisms are used and combined in our CasRedSCAN. We analyzed the effect of these two attention mechanisms in our CasRedSCAN. The result is illustrated in Table III and evaluated using AAPM dataset. We compared our CasRedSCAN’s performance with or without channel attention or spatial attention. As we can observe, both channel attention and spatial attention can improve the reconstruction performance, and the combination of both attentions provides the best performance with the least variation, and significantly outperforms the baseline CasRedSCAN without both channel and spatial attentions.

#### 3) Sinogram Evolution:

**Fig. 8.**
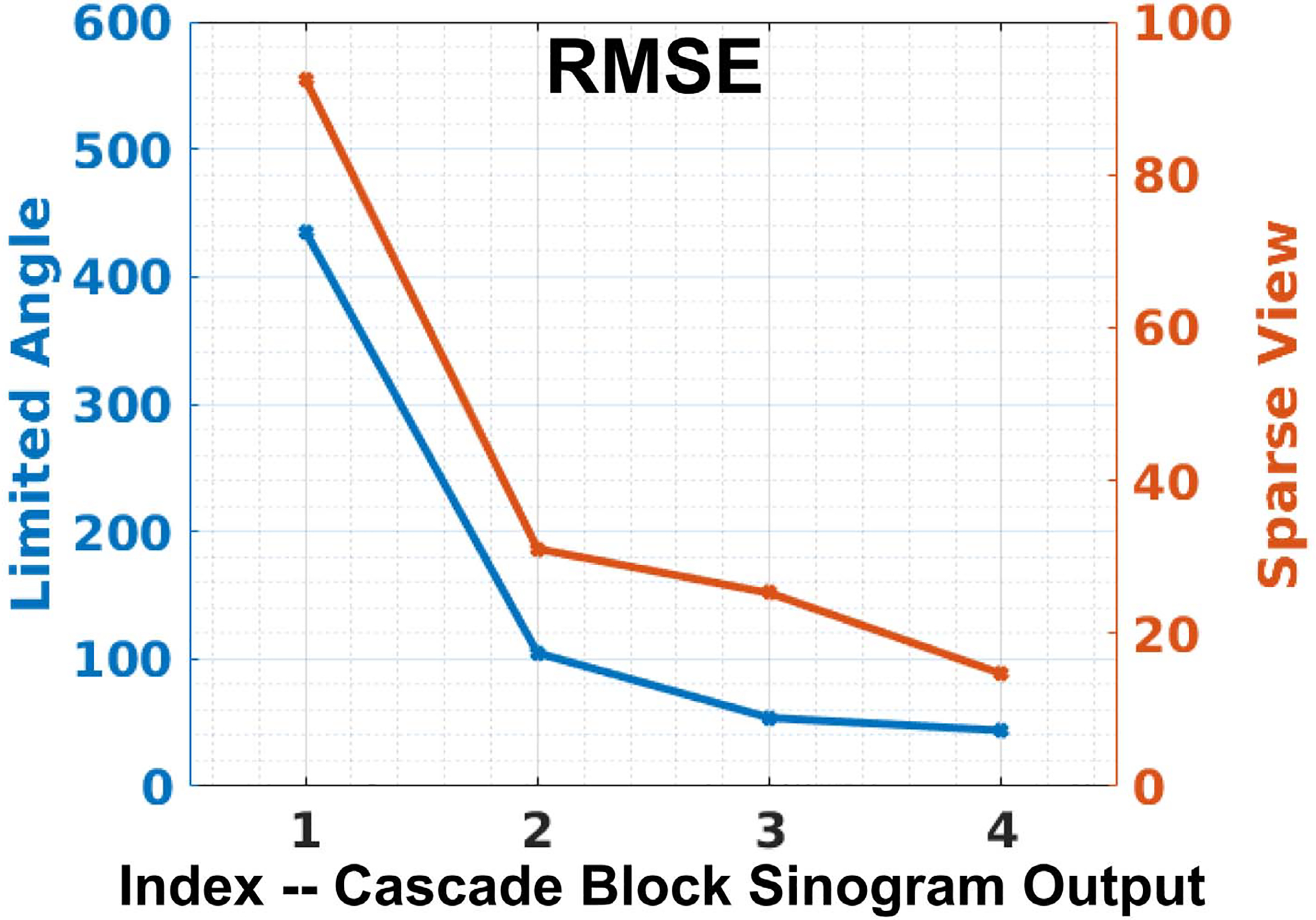
Sinogram errors over the cascade block’s output in our CasRedSCAN for **limited angle reconstructions** (120° limited angle) and **sparse view reconstructions** (1/4 downsampling).

With the number of cascade block set to 4 in our CasRedSCAN, we further analyzed how the generated sinogram evolves over the cascaded network. We computed the mean RMSE between each cascade block’s sinogram outputs and the ground truth full view sinogram. The results for both LA and SV are plotted in Figure 8. As we can see, the sinogram errors gradually reduce as the generated data passes through the next cascaded block, while the rate of sinogram error reduction starts to converge after the first cascade block.

#### 4) PDFL Parameter:

**Fig. 9.**
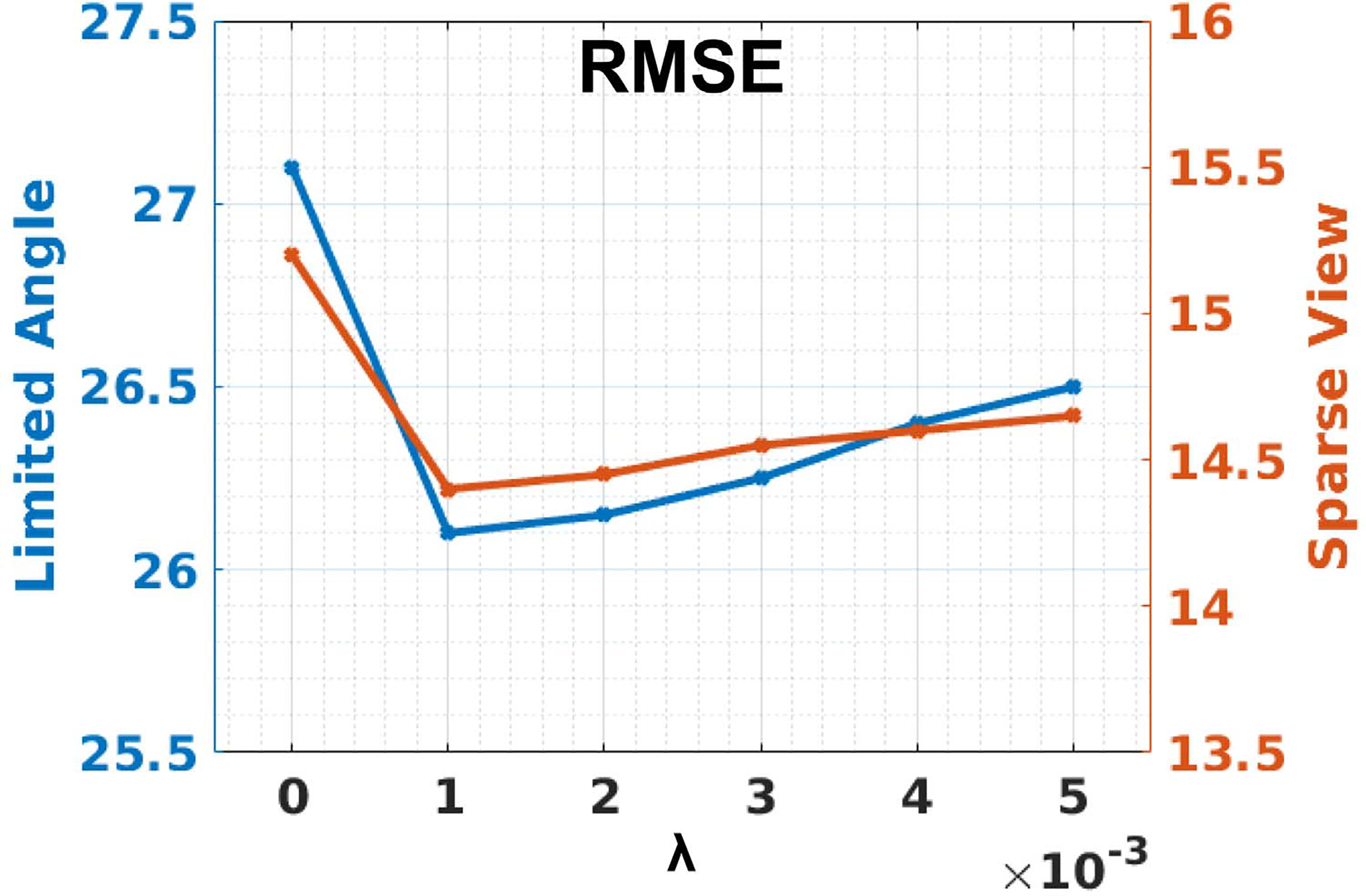
Impact of *λ* in PDFL for **limited angle reconstructions** (120° limited angle) and **sparse view reconstructions** (1/4 downsampling).

In PDFL, *λ* is the noise level parameter that controls the linear combination of the acquired projection data and the projection data of RedSCAN’s output. Assuming low noise x-ray acquisition as in our experiments, *λ* should be a small value as the impact of noise is minimal. We analyzed the impact of *λ* under both LA and SV conditions. The results are summarized in Figure 9. As we can observe, reconstruction without considering the noise, i.e. *λ* = 0, leads to degradation on reconstruction performance. Setting *λ* = 0.001 leads to the best reconstruction performance in our search range, while the RMSE difference is less than 1 between *λ* = 0.001 and *λ* = 0.005.

#### 5) Embedded Networks:

**Fig. 10.**
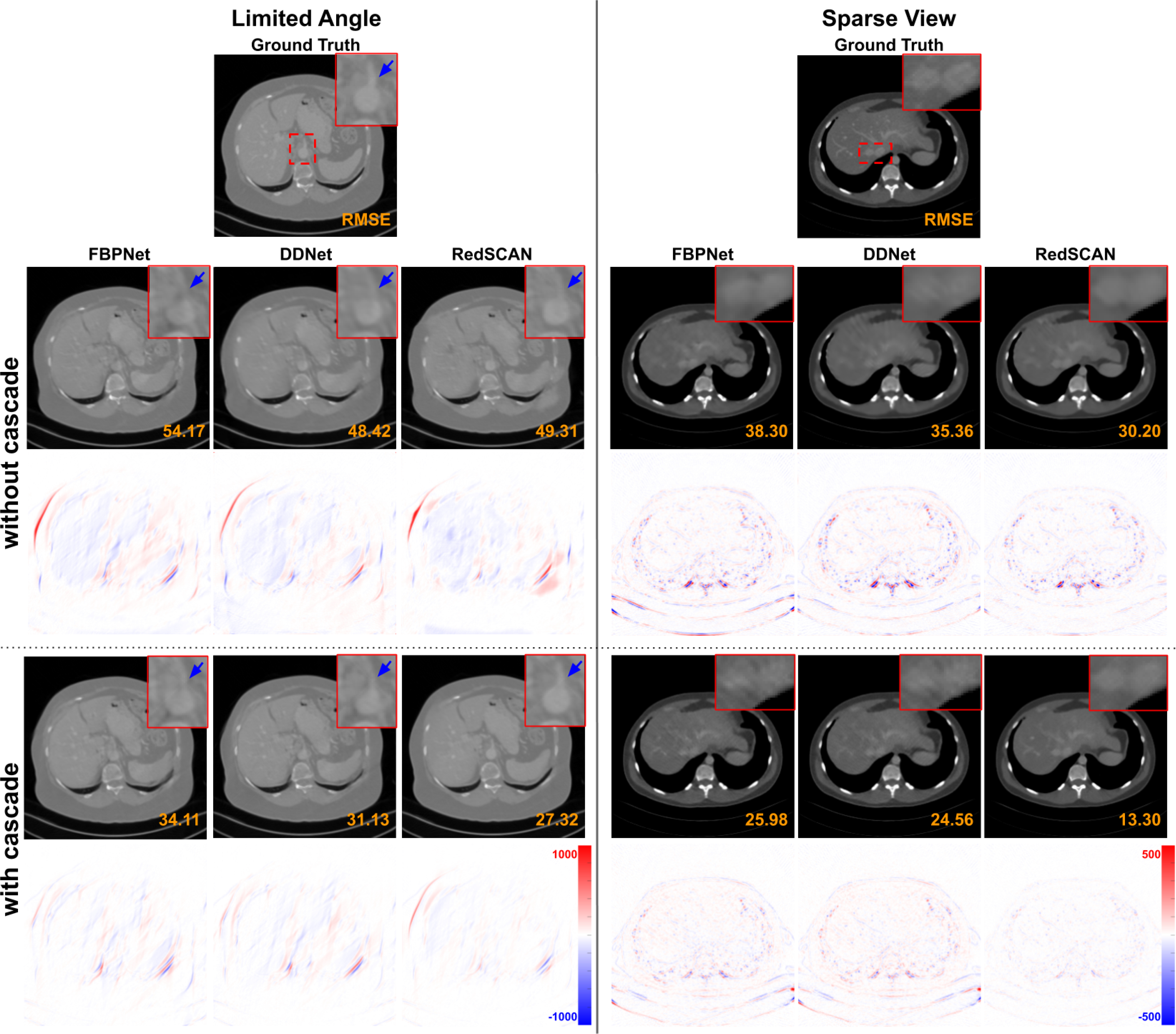
Comparison of **limited angle reconstructions** (120° limited angle) and **sparse view reconstructions** (1/4 downsampling) with and without cascade framework using different basic networks. The display window of limited angle reconstruction is [−1000 1000] HU. The display window of sparse view reconstruction is [−300 800] HU.

We embedded different previous image-to-image reconstruction networks [13], [18], [19] into our cascaded network and compared the performance with or without cascade. The qualitative results are visualized in Figure 10. The quantitative results are summarized in Table IV. The number of cascade is set to 4 in this study. As we can observed, embedding different previous image-to-image networks into our cascade design improves the reconstruction performance, while RedSCAN embedded into our cascade network achieves the best reconstruction performance.

## VI. Discussion

In this paper, a novel reconstruction framework, named CasRedSCAN, is proposed. Inspired by the recent advances in image super-resolution network designs and the projection data constraint in MBIR, we designed a customized RedSCAN as our backbone image reconstruction network, and we built a projection data fidelity layer that can be embedded in deep networks. First of all, our RedSCAN is developed based on image super-resolution network [35] with an addition of spatial-channel attention, which allows our RedSCAN to re-calibrate the channel attention and gives different levels of attention on recovering texture details at different spatial locations, as artifact distribution is not uniform in the image. In fact, Hu *et al.* [36] recently also demonstrated that spatial-channel attention can boost the image super-resolution performance. Then, we develop PDFL that can be concatenated to the RedSCAN’s cascade outputs to ensure the projection data fidelity at the sampled projection views. Our PDFL based on the analytical FBP solution with fan-beam geometry allows it to be embedded in a deep network and used during training and inference.

We demonstrate the feasibility of our CasRedSCAN on both LA and SV tomographic reconstruction tasks, as shown in the result section. Firstly, the LA acquisition is more difficult to reconstruct as compared to the SV acquisition since a range of projection angles are not covered in the LA acquisition. Severe image artifacts at these projection angles can be observed when using conventional FBP. As a result, the general performance of LA reconstructions are inferior to the SV reconstruction performance. For example, in 120° LA reconstruction, while previous methods can mitigate the artifacts and recover PSNR up to 37.94 and SSIM up to 0.970, they still have difficulties in recovering the organ boundaries that are critical for clinical diagnosis and treatment planning. Our CasRedSCAN provides superior reconstructions with clear organ boundaries and is able to improve the PSNR to 41.48 and SSIM to 0.983. In 1*/*4 SV reconstruction, while previous methods can generate visually plausible image content, the reconstruction prediction without projection data fidelity can result in artificial texture which is undesirable in clinical tasks. Our CasRedSCAN with PDFL can better preserve the image fidelity by incorporating the already-sampled projection data, resulting in best performance in terms of PSNR, SSIM, and RMSE.

Furthermore, we demonstrate the feasibility of our CasRedSCAN on CT lesion imaging under LA and SV conditions. Lesion is highly heterogeneous, and CT is one of the primary tool for diagnosis. Obtaining high-quality lesion region reconstruction under LA and SV is essential for disease diagnosis, staging, as well as planning and evaluation of treatment. While previous methods can reduce the reconstruction artifacts from the whole image perspective, the reconstruction in lesion region with high heterogeneity is still unsatisfying - the lesion boundary and texture are highly distorted by previous methods which will negatively impact the subsequent treatment options. On the other hand, our CasRedSCAN can better preserve the lesion reconstruction even the lesions are highly heterogeneous. For example, the supplying vessels of LA lung lesion in Figure 5 are totally missed by previous methods, while our CasRedSCAN can better recover it. The complex interior texture of SV lung lesion in Figure 5 is highly distorted by previous methods, but our CasRedSCAN can still preserve the structure. In Figure 5, liver and kidney lesions embedded in soft-tissue background with low contrast are prone to smooth-out in SV and distorted in LA by previous methods, and our CasRedSCAN can better recover the boundary and the contrast of the lesions.

We believe there are several reasons that potentially lead to the superior performance of using RedSCAN in CasRedSCAN. First of all, our RedSCAN has no image downsampling for abstraction, thus keeping the image restoration on original resolution. Second, convolutional layers in different depths have different sizes of receptive fields, resulting in hierarchical features. Image restoration should utilize all the hierarchical features, instead of only the last layer output. Our RedSCAN concatenating all the hierarchical features can potentially better learn the restoration. Thirdly, the hierarchical features are generated by our residual dense channel-spatial block that allows better feature learning at each hierarchical level. Moreover, the residual connection in each block also allows the gradient to be better passed to earlier layers, thus helping the training of our wide network design. As shown in Table , the design of our RedSCAN also provides a relatively smaller amount network parameter (0.51M) as compared to the previous method. Specifically, the RedSCANs in CasRedSCAN share the same network parameter and there is no learnable parameter in PDFL, thus the CasRedSCAN’s parameter size remains the same as RedSCAN regardless of the number of cascading. In this case, our CasRedSCAN using the least amount of parameters achieves the best limited view reconstruction performance.

The presented work also has potential limitations. First of all, the inference time is longer compared to the previous deep learning based methods, as illustrated in Table VI. This is caused by the cascaded design with PDFL interleaved. On one hand, the iterative reconstruction prediction will increase the computation time. On the other hand, even though FBP is a fast analytic solution, the forward projection and FBP operations in PDFL still consume computation times. The combination of these two results in longer training and inference time. However, the inference time is about 150 ms which is acceptable and much faster than previous MBIR methods. Moreover, in our PDFL, we assume 360 degrees fan-beam projection combined from the already sampled sinogram and the predicted sinogram. The minimal complete sinogram with reduced number of projection could reduce the computation time of PDFL. However, additional step of sinogram weighting, such as Parker weighting [37], could be incorporated to address the data redundancy issue. Secondly, while increasing the number of cascade block in CasRedSCAN improves the performance, the memory consumption will increase along with longer training and inference time. As illustrated in Figure 7, the increase in performance starts to converge after *n* = 3. Thus, in this work, we set *n* = 4 to balance the memory consumption and inference time of our CasRedSCAN.

The architecture of our CasRedSCAN also suggests several interesting topics for future studies. The first one is combining the projection data fidelity layer with the deep learning based radon inversion techniques [38]. The cascaded framework with projection data fidelity can provide the projection domain constraint during the radon inversion via deep learning. It can potentially improve the inversion stability, yielding reconstruction with better data fidelity. Secondly, given the superior lesion region reconstruction performance demonstrated in the result sections, our framework could also potentially improve the projection data based Computer-Aided Diagnosis (CAD). Recently, there are increasing interests on combining limited-view reconstruction and CAD for a joint reconstruction-CAD network structure, and improved CAD performance is expected with such an end-to-end training strategy [39], [40]. We believe that our CasRedSCAN with high-quality lesion region reconstruction would provide new opportunities for these kinds of studies. Thirdly, CT metal artifact reduction (MAR) under limited-view acquisition is an important research direction. Current MAR techniques are mostly limited to full-view acquisition [41], [42]. The current state-of-the-art metal artifact reduction algorithm, such as DuDoNet [41], utilizes projection space and image space simultaneously which is similar to our CasRedSCAN design. Our CasRedSCAN could potentially integrated with current MAR network for MAR under limited view conditions. Fourthly, low-dose CT combined with limited-view acquisition may further reduce the radiation dose. As a matter of fact, Shan *et al.* [43] and Wu *et al.* [44] had proposed cascaded network structures with basic network of UNet [15] or sequential CNN layers, and demonstrated their efficiency in low-dose CT. As cascade network is also potentially efficient in low-dose CT, our CasRedSCAN could be adapted to limited-view low-dose CT that may further reduce the radiation dose and acquisition time. Lastly, we believe our CasRedSCAN could be adapted to other tomography imaging modalities with similar applications, such as SPECT, PET, and Cryo-ET [45]–[47].

## VII. Conclusion

In this work, we proposed a cascaded network with RedSCAN and PDFL, a novel framework for limited view tomographic reconstruction. The proposed PDFL is interleaved in our cascaded network to ensure the sampled sinogram is consistent in sinogram domain with the network cascaded output. A customized image restoration network is used as the backbone in the cascaded network. Comprehensive evaluation demonstrates that our CasRedSCAN can provide high-quality limited angle and sparse view tomographic reconstruction while reducing radiation dose and shortening scanning time.

## Figures and Tables

**TABLE I:** Quantitative Comparison of **Limited Angle Reconstruction** and **Sparse View Reconstruction** Results Under Different Limited Angle and Sparse View Settings Using PSNR (dB), SSIM, and RMSE on AAPM Dataset. Best Results Are Marked in red

PSNB/SSIM/RMSE	Limited Angle Reconstruction			Sparse View Reconstruction			Time (ms)	Number of Parameters
	90°	120°	150°	1/6	1/4	1/2		
FBP	17.76/.555/388.7	21.76/.693/246.3	26.81/.782/138.5	26.85/.513/137.8	30.19/.648/94.1	39.02/.896/33.9	2.3	-
TV [6]	22.56/.762/230.3	30.67/.875/132.1	33.74/.898/Ó9.7	30.91/.895/70.3	34.13/.911/41.3	35.83/.934/18.7	3096.3	-
EBPNet [13]	28.66/.887/111.7	35.14/.959/53.6	40.80/.982/28.2	34.73/.933/55.2	39.26/.962/32.8	46.61/.986/14.1	7.2	30M
DDNet [18]	31.03/.921/85.6	36.49/.965/45.9	41.45/.988/25.9	35.07/.933/53.3	39.60/.963/31.7	45.70/.984/15.6	5.1	0.56M
FUNet [19]	30.27/.903/93.5	35.87/.960/48.3	41.01/.985/26.7	35.01/.933/54.8	39.52/.962/32.0	46.64/.986/14.0	5.6	36M
CTNet [1]	29.05/.889/106.8	35.33/.962/52.4	40.97/.984/27.5	35.81/.936/48.8	39.80/.963/30.9	46.73/.987/13.8	10.3	31M
DCAR [21]	30.25/.900/94.4	37.94/.970/39.1	43.87/.989/21.7	36.99/.948/42.6	41.18/.973/26.3	47.01/.989/11.5	3187.6	30M
CasRedSCAN	34.74/.952/56.42	41.48/.983/26.1	48.23/.995/11.8	43.13/.979/21.1	46.43/.989/14.4	51.66/.996/7.8	148.2	0.51M

**TABLE II:** Quantitative Comparison of **Limited Angle Reconstructions** (120° Limited Angle) and **sparse view reconstructions** (1/4 Downsampling) Results Using PSNR (dB), SSIM, and RMSE. Best Results Are Marked in red

**LA**	Bone	Abdomen	Mediastinum	Liver	Lung	Kidney	Soft Tissue	Pelvis
FBP	22.29/.652/231.	21.83/.675/244.	22.54/.691/225.	21.73/.660/247.	22.18/.627/234.	21.91/.681/241.	22.81/.696/219.	22.75/.699/219.
TV [6]	30.67/.877/130.	30.18/.871/136.	30.83/.877/129.	30.27/.868/134.	30.64/.875/132.	30.34/.871/140.	31.03/.880/128.	30.83/.878/129.
FBPNet [13]	36.68/.945/45.6	39.99/.969/30.6	36.52/.956/45.7	38.47/.964/37.6	35.11/.932/53.9	40.47/.972/28.7	37.71/.961/41.0	38.01/.967/38.7
DDNet [18]	38.86/.971/36.9	41.87/.982/25.2	38.25/.972/38.7	40.67/.979/29.9	36.88/.963/44.5	42.97/.985/22.1	39.97/.977/33.0	40.15/.981/31.0
FUNet [19]	36.93/.948/43.2	40.14/.971/29.9	36.78/.960/42.9	38.93/.971/33.8	35.22/.943/49.1	41.06/.979/26.7	38.03/.968/39.9	38.04/.969/38.1
CTNet [1]	37.13/.949/40.8	40.11/.971/30.2	37.33/.962/41.7	38.97/.972/32.4	35.91/.952/46.2	41.17/.979/24.3	37.96/.963/40.2	38.00/.967/38.4
DCAR [21]	39.32/.977/32.5	42.92/.984/21.3	39.11/.976/33.8	40.86/.980/28.5	37.69/.970/41.3	43.13/.986/20.6	40.33/.980/30.5	40.84/.982/29.8
CasRedSCAN	42.08/.984/25.2	45.59/.990/16.2	41.83/.985/25.6	43.88/.987/20.7	40.72/.981/28.7	46.36/.991/14.7	43.34/.988/22.4	43.52/.989/20.8
**SV**	Bone	Abdomen	Mediastinum	Liver	Lung	Kidney	Soft Tissue	Pelvis
FBP	28.71/.591/112.	31.01/.676/85.3	29.50/.600/101.	30.83/.667/87.1	27.23/.538/132.	31.37/.680/81.7	29.94/.617/97.3	30.35/.636/91.8
TV [6]	32.31/.899/48.2	35.62/.919/39.4	33.94/.907/44.6	34.16/.911/41.8	31.54/.897/47.9	35.73/.918/39.5	33.98/.911/41.2	34.08/.910/42.5
FBPNet [13]	38.89/.952/35.5	42.28/.973/23.4	39.37/.961/32.9	41.91/.972/24.6	36.80/.931/45.6	42.56/.975/22.6	40.50/.968/29.3	41.61/.974/25.2
DDNet [18]	40.93/.960/28.5	44.67/.980/18.0	41.17/.968/27.1	44.23/.980/19.0	38.60/.941/37.2	45.12/.982/17.0	42.76/.974/22.9	44.13/.980/19.0
FUNet [19]	38.95/.956/33.1	42.83/.978/20.9	39.87/.966/31.2	42.37/.977/21.3	37.01/.938/42.5	42.88/.977/20.1	40.82/.970/27.8	41.68/.974/24.6
CTNet [1]	38.96/.956/32.8	42.88/.978/20.5	39.97/.968/29.7	42.20/.973/22.8	37.12/.940/40.2	42.99/.979/19.9	40.79/.968/28.7	41.73/.976/23.3
DCAR [21]	42.22/.972/23.6	45.13/.982/17.1	42.52/.973/23.5	45.79/.981/18.6	39.77/.958/32.9	45.54/.982/16.5	43.32/.977/19.2	45.67/.982/17.7
CasRedSCAN	46.00/.987/15.8	49.80/.994/9.8	46.71/.990/14.3	49.59/.994/10.2	43.90/.981/20.1	50.11/.994/9.5	48.24/.990/12.6	49.48/.994/10.2

**TABLE III:** Attention Mechanism Analysis Using PSNR, SSIM, RMSE. ✓ and ✘ Means Channel Attention (CA) and Spatial Attention (SA) Used and Not Used in Our CasRedSCAN. The Optimal Results Are in Bold.

Task	CA	SA	PSNR	SSIM	RMSE
	✘	✘	39.61 ± 1.78	.973 ± .010	30.7 ± 4.3
LA	✓	✘	40.98 ± 1.62^†^	.979 ± .007^†^	28.8 ± 4.0^†^
	✘	✓	40.93 ± 1.63^†^	.978 ± .008^†^	28.6 ± 4.0^†^
	✓	✓	41.48 ± 1.51*	.983 ± .005*	26.1 ± 3.8*

	✘	✘	44.01 ± 1.38	.979 ± .009	18.8 ± 2.8
SV	✓	✘	45.49 ± 1.23^†^	.983 ± .006^†^	16.9 ± 2.3^†^
	✘	✓	45.35 ± 1.24^†^	.981 ± .005^†^	16.7 ± 2.4^†^
	✓	✓	46.43 ± 1.05*	.989 ± .002*	14.4 ± 1.7*

* Means the Difference Compared to Baseline Without SA and CA Are Significant at p < 0.1

While † Means Not Significant

**TABLE IV:** Quantitative Comparison of **limited angle reconstruction** (120°) AND **SPARSE VIEW RECONSTRUCTION** (1/4 DOWNSAMPLING) Results Using Different Networks With and Without Our Cascaded Framework

SSIM/RMSE-LA	FBPNet	FUNet	DDNet	Ours

Single	.959/53.6	.960/48.3	.965/45.9	.966/47.7
With Cascade	.970/39.9	.973/36.3	.978/32.5	.983/26.1

SSIM/RMSE-SV	FBPNet	FUNet	DDNet	Ours

Single	.962/32.8	.962/32.0	.963/31.7	.967/27.8
With Cascade	.977/23.8	.978/22.8	.981/19.3	.989/14.4

## Footnotes

1 implement with Pytorch using torch.nn.functional.grid_sample

2 implement with Pytorch using torch.fft and torch.ifft

3 implement with Pytorch’s Custom C++ and CUDA extensions

4 http://pytorch.org/
